# Human Theta Burst Stimulation Combined with Subsequent Electroacupuncture Increases Corticospinal Excitability

**DOI:** 10.1155/2020/8824530

**Published:** 2020-12-23

**Authors:** Jiali Li, Meng Ren, Wenjing Wang, Shutian Xu, Sicong Zhang, Yuanli Li, Chunlei Shan

**Affiliations:** ^1^Center of Rehabilitation, Yueyang Hospital of Integrated Traditional Chinese and Western Medicine, Shanghai University of Traditional Chinese Medicine, Shanghai, China; ^2^School of Rehabilitation Science, Shanghai University of Traditional Chinese Medicine, Shanghai, China; ^3^Engineering Research Center of Traditional Chinese Medicine Intelligent Rehabilitation, Ministry of Education, Shanghai, China

## Abstract

**Objective:**

Intermittent theta burst stimulation (iTBS) is a widely used noninvasive brain stimulation for the facilitation of corticospinal excitability (CSE). Previous studies have shown that acupuncture applied to acupoints associated with motor function in healthy people can reduce the amplitude of the motor-evoked potentials (MEPs), which reflects the inhibition of CSE. In our work, we wanted to test whether the combination of iTBS and electroacupuncture (EA) would have different effects on CSE in humans.

**Methods:**

A single-blind sham-controlled crossover design study was conducted on 20 healthy subjects. Subjects received 20 minutes' sham or real EA stimulation immediately after sham or real iTBS. MEPs, short-interval intracortical inhibition (SICI), intracortical facilitation (ICF), cortical silent period (CSP), and central motor conduction time (CMCT) were recorded before each trial, and immediately, 20 minutes, and 40 minutes after the end of stimulation.

**Results:**

In the sham iTBS group, EA produced a reduction in MEPs amplitude, lasting approximately 40 minutes, while in the real iTBS group, EA significantly increased MEPs amplitude beyond 40 minutes after the end of stimulation. In sham EA group, the recorded MEPs amplitude showed no significant trend over time compared to baseline. Among all experiments, there were no significant changes in SICI, ICF, CSP, CMCT, etc.

**Conclusion:**

These data indicate that immediate application of EA after iTBS significantly increased corticospinal excitability. This trial was registered in the Chinese Clinical Trial Registry (registration no. ChiCTR1900025348).

## 1. Introduction

In 2005, Huang et al. proposed theta burst stimulation (TBS) as a special paradigm of repetitive transcranial magnetic stimulation (rTMS) which mimics endogenous theta oscillation and is able to modulate human brain excitability beyond the time of stimulation [1]. TBS was originally developed by observing the pattern of neuronal firing that occurred during the exploration of rats and was able to modulate brain activity beyond the time of stimulation in humans [2]. TBS mimics the combination of a 100 Hz gamma frequency and a 5 Hz theta frequency and induces obvious long-term potentiation (LTP) in rat hippocampal slices [3]. This patterned stimulation protocol was adapted in humans using similar frequency parameters to animal models and has been widely used for over a decade. Typically, TBS in humans involves the application of high-frequency bursts (3 pulses at 50 Hz) at low-frequency interval (5 Hz) using a total of 600 pulses at 70∼80% of active/resting motor threshold (a/rMT). When applied continuously (cTBS) for 40 s, TBS has shown to change corticospinal excitability (CSE) measured via motor-evoked potentials (MEPs) for up to at least 20 min. When applied intermittently (iTBS; 2 s on, 8 s off) for 192 s, an opposite effect was observed up to 30 min [1].

Many studies used the amplitude of MEPs recorded from peripheral muscles, which is widely used to evaluate CSE, to assess the effect of iTBS, and have demonstrated that iTBS increases cortical excitability in healthy individuals beyond 20 min after stimuli [4, 5]. This kind of increase is thought to be associated with long-term potentiation (LTP), which plays an important role in learning [6, 7]. In addition to MEPs, various TMS paradigms are also used to evaluate the state of neural circuits, and potentially revealing the underlying mechanism for the interventions and treatments, such as short‐interval intracortical inhibition (SICI) and intracortical facilitation (ICF) [8]. MEPs reflects the overall excitability of the cortex, spinal, and corticospinal [9]. SICI refers to the phenomenon that a subthreshold conditioning stimulation (CS) suppresses the MEP induced by subsequent suprathreshold test stimulation (TS) at interstimulus intervals (ISIs) of 1–5 ms. SICI is the most common and well‐studied intracortical circuits in the primary motor cortex (M1) [10]. ICF is assessed at an ISI of 10 ms. The CS intensities usually ranged from 75 to 95% AMT in different individuals to produce consistent test MEP facilitation [11].

Acupuncture is an important part of traditional Chinese medicine. Electroacupuncture (EA) combines acupuncture and electrical stimulation, which is widely welcomed worldwide due to its standardization and repeatability. Compared with manual acupuncture, electroacupuncture is more effective because of its more standardized parameters, higher repeatability, wider range of stimulation, and lower demands on acupuncturists [12]. Previous studies have proved that acupuncture suppresses corticomotor excitability of healthy individuals [13–16]. However, the underlying mechanisms and efficacy of acupuncture on brain function remain confused, commonly hindered by low-quality study designs. In this study, four most frequently used acupoints in treatment of dyskinesia after stroke, Quchi (LI11), Hegu (LI4), Zusanli (ST36), and Yanglingquan (GB34), are chosen to be studied [17].

In the present study, we used single TMS to measure resting motor threshold (RMT), active motor threshold (AMT), MEP, CSP, CMCT, and paired pulse TMS to measure SICI and ICF to determine whether combination of iTBS and EA could modulate the neural excitability of the M1 in healthy adults when compared with only iTBS, electroacupuncture, or none. We hypothesized that combination of acupuncture and iTBS could increase motor cortical excitation and reduce motor cortical inhibition.

## 2. Methods

### 2.1. Subjects

22 healthy, right‐handed subjects were recruited for participation in the current study via recruitment advertisements. One subject dropped out in the experiment process, while one was excluded because of taking antidepressants. Finally, a total of 20 subjects (22.8 ± 3.09 years, 11 females) were included in the statistical analysis. All subjects were right-handed, as assessed by the Edinburgh Handedness Inventory [18], without taking any regular drugs (recreational or clinically indicated), and no contraindication to TMS, and no history of neurological or psychiatric disorders [19]. All subjects provided written informed consent prior to experimentation. This experimental procedure was approved by the local ethics committee of Yueyang Hospital of Integrated Traditional Chinese and Western Medicine. All experiments conformed to the declaration of Helsinki. This trial was registered in the Chinese Clinical Trial Registry (registration no. ChiCTR1900025348).

### 2.2. Transcranial Magnetic Stimulation

TMS was delivered by two Magstim 200 stimulators connected via a Bistim module to a figure-of-eight coil (double-circular-70 mm coil) (Magstim 2002, Magstim Co., UK), held with the handle pointing posterolateral. Ag/AgCl surface electrodes in a belly-tendon montage were used to record electromyography (EMG) from the right first dorsal interosseous (FDI) muscle. EMG signals were amplified, digitized, band-pass-filtered (20 Hz-10 KHz), sampled, and saved to a disk for offline analysis (Keypoint, Dantec, Denmark).

The approximate position of the left M1 is C3 point in the international 10/20 EEG positioning system. The “hot spot,” i.e., the location on the scalp with the largest and most consistent MEP, was found using manually triggered single-pulse TMS (sp-TMS). Resting motor threshold (RMT) was defined as the lowest stimulus intensity required to evoke MEPs with peak-peak amplitudes of ≥50 *μ*V in the relaxed FDI in 5 out of 10 consecutive trails [20]. When subjects maintained the voluntary contraction of FDI muscle at 20% of the maximum strength, active motor threshold (AMT) was defined as the lowest stimulus intensity required to evoke MEPs with peak-peak amplitudes of ≥200 *μ*V in 5 out of 10 consecutive trials [20]. Use 2 msec interstimulus interval (ISI) to measure SICI, and ISI of 10 msec to measure ICF. The conditioning stimulus was 80% of RMT and the test stimulus was 120% MT1 mV [21]. Both SICI and ICF were calculated by averaging of the peak-peak amplitude for 15 consecutive tests. Cortical silent period (CSP) was measured with single pulses applied at RMT, with subjects pinching their right thumbs and forefingers together at 20% of their maximum voluntary contraction which was visualized by electromyography waveform recorded in real time. In a single trial, CSP was measured as the time from the onset of MEP until the recurrence of voluntary EMG activity. Mean CSP duration was calculated from 15 consecutive trials [20]. CMCT was calculated by subtracting the conduction time in the peripheral nerve obtained by magnetic stimulation of the cervical root (peripheral motor latency) from the MEP cortical latency. A total of 15 pairs of trials each were recorded, and mean CMCT was calculated from them. All experiments were completed in a quiet and well-shielded room, with subjects relaxed and alert.

We used iTBS as introduced by Huang and colleagues [1]. iTBS was performed with the MC-B70 Butterfly “8” shaped coil (MagPro X100, MagVenture, Denmark). The coil was placed tangentially to the subject's scalp so that the midpoint of the coil is aligned with the “hot spot” position, with an angle of 45° from the midline. Theta burst stimulation (TBS) contains of a burst of 3 stimuli at 50 Hz, which was repeated at intervals of 200 msec. In the iTBS protocol, 2 seconds' TBS trains (30 pulses) were repeated every 10 seconds for 190 s, with a total number of 600 pulses. The stimulus intensity was 80% AMT. We used one of the most reliable methods reported in previous studies to implement the sham stimuli, with the figure of “8” coil placed vertically on the scalp, and the edge of the coil in contact with the “hot spot” position on the scalp [22]. The parameters of sham iTBS were set the same as real iTBS protocol.

### 2.3. Electroacupuncture

An experienced acupuncturist performs the electroacupuncture intervention. Real EA was applied to right extremities at acupoints of Quchi (LI11), Hegu (LI4), Zusanli (ST36), and Yanglingquan (GB34) using disposable acupuncture needles (0.30 × 40 mm, Hwato, Suzhou, China) and hollow foam pads attached to the skin. Subjects were asked to close their eyes during inserting needles. When subjects had a sense of Deqi, the needles were connected to electroacupuncture apparatus (Hwato, Suzhou, China) with a 5 Hz continuous wave for 20 minutes, while sham EA was delivered using custom blunt needles (0.30 × 25 mm, Hwato, Suzhou, China), which gave the skin a tingling sensation without penetrating into the skin, standing on the acupoints with foam pads. Piercing against the foam pad gave the subjects a sense of acanthesthesia similar to real acupuncture. The electroacupuncture apparatus was modified so that there was no current out and the subjects were told that the apparatus was in low-frequency low-intensity mode.

### 2.4. Experimental Design

The effect of iTBS in combination with EA on corticospinal excitability was assessed using a randomized, single-blinded, sham-controlled, crossover study design. Subjects attended 4 sessions: (1) sham iTBS + sham EA; (2) sham iTBS + real EA; (3) real iTBS + sham EA; (4) real iTBS + real EA. The order of sessions was counter-balanced across the 4 groups, and the carryover possibility was reduced by separating each session by at least 5 days. Use MATLAB 2013 software to achieve randomization of the intervention sequence. The combination of the four intervention methods has a total of 24 permutations. Use MATLAB to generate two random numbers between 1 and 24, and remove the corresponding permutations from the 24 permutations. The 22 subjects were randomly numbered, the numbers from 1 to 22 were randomly arranged using MATLAB, and the subjects were randomly assigned to the 22 sorting orders according to this set of numbers.

**Figure 1:**
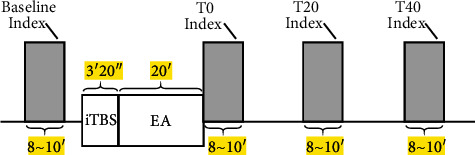
Experimental design. Before intervention (baseline), electrophysiological indexes (RMT, AMT, MT1 mV, MEP_MT1 mV_, SICI, ICF, CSP, CMCT) were measured and recorded. The stimulation included 192 sec iTBS session (sham or real), followed by 20 min EA session (sham or real). Electrophysiological indexes were recorded immediately after the end of stimulation and 20 and 40 minutes later.

The experimental outline is shown schematically in Figure 1. Outcome indicators such as MEP were measured prior to each trial (baseline), and then real or sham iTBS was delivered, and real or sham EA intervention was applied immediately after iTBS. After the intervention of EA, outcome indicators measurements were performed immediately and every 20 minutes until 40 minutes (T0, T20, and T40).

Studies have shown that the after-effects of iTBS vary significantly among different subjects. The reasons for this variability are varied; there were many physiological factors contributing to this variability in response to iTBS, such as age, gender, handedness, genetics, quality of sleep and arousal, the state of motor system activation, and intake of caffeine, nicotine, alcohol, antidepressants, benzodiazepines, and antiepileptic drugs, and so on [23]. The experimental outline is illustrated in Figure 1. iTBS (real or sham) was delivered first after baseline MEP measurement and EA (real or sham) was delivered immediately after iTBS. Measurements of outcome indicators were performed immediately after EA and every 20 minutes until 40 minutes (T0, T20, and T40).

In this study, the electrophysiological index of right hand FDI (MEP_MT1 mV_) was used as the main outcome index. When at least 5 MEP peak-peak amplitude of MEP recorded in 10 consecutive single pulse magnetic stimuli is ≥1 mV, the threshold value of stimulate intensity is denoted as MT1 mV. MT1 mV was measured before intervention (baseline). MEP at 4 time points before and after the intervention (baseline, T0, T20, T40) was measured using MT1 mV at baseline (MEP_MT1 mV_); i.e., MEP at different time points was assessed using a fixed stimulus intensity to evaluate the difference of cortical excitability at each time point. At each time point, 15 MEPs were recorded continuously, and the mean value of peak-to-peak amplitude was calculated during offline processing. SICI, ICF, CMCT, and CSP were secondary outcome indicators.

### 2.5. Data Analysis and Statistics

In the 15 trials recorded at each time point, peak-to-peak amplitudes or CMCT was measured using a custom-made MATLAB script (MATLAB 2013b, The MathWorks, Inc., Natick, MA, USA). Average MEP amplitude was calculated and converted to logarithmic value (LOG MEP_MT1 mV_) to make the data follow a normal distribution. Do the same conversion for other indicators such as SICI, etc.

Three-factorial repeated-measures analysis of variance (rm-ANOVA) was used to analyze the changes in outcome indicators, including three factors: iTBS (“real,” “sham”) and EA (“real,” “sham”) and time (pre-stimulation, 0, 20, and 40 min after stimulation). Post hoc paired *t*-tests were used to examine differences from baseline and differences between values at every time point (Bonferroni-corrected for multiple comparisons). *P* < 0.05 was considered to be statistically significant. The SPSS 21.0 software was used for statistical analysis.

## 3. Results

**Figure 2:**
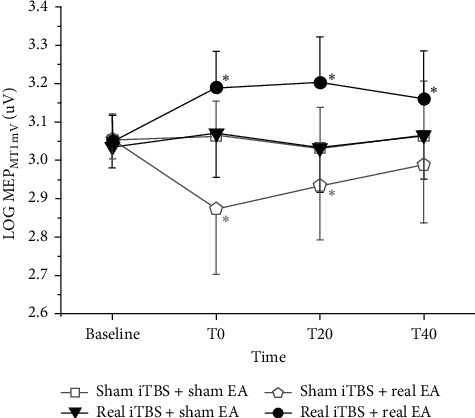
LOG MEP_MT1 mV_ with different interventions. There was no significant difference between 4 conditions at baseline. And the MEP_MT1 mV_ amplitude at different time points differs across the conditions. With sham EA, the MEP_MT1 mV_ amplitude did not vary significantly with time points. The condition of sham iTBS and real EA suppressed the MEP_MT1 mV_ amplitude significantly, and the condition of sham iTBS and real EA improved the MEP_MT1 mV_ amplitude significantly.  ^*∗*^There is a significant difference from the baseline, *P* < 0.01.

RMT and AMT at baseline of the four conditions are listed in Table 1, and there is no significant difference between the conditions (Bonferroni-corrected pairwise *t*-test). The amplitudes of MEP_MT1 mV_ of all subjects are plotted in Figure 2. A 2-way ANOVA of LOG MEP_MT1 mV_ with main factors of iTBS and EA at baseline showed no difference between the 4 experimental conditions (interaction: *F* (1, 19) = 0.185, *P* = 0.672; main effect of iTBS: *F* (1, 19) = 0.954, *P* = 0.341; main effect of EA: *F* (1, 19) = 0.346, *P* = 0.563). This result indicated that the cortical excitability of the subjects was similar at the start of every session.

As shown in Table 2, a three-way ANOVA with main factors of iTBS, EA, and time showed a strong interaction between three factors (*F* (3, 57) = 11.019, *P* < 0.001), indicating that the MEP_MT1 mV_ amplitude at different time points differs across the conditions. Two-way ANOVAs with main factors of iTBS or time were performed, respectively, in the conditions of real EA and sham EA. The results indicated that, in the real EA condition, there was significant interaction between iTBS and time (*F* (3, 57) = 23.484, *P* < 0.001). So, the presence of EA resulted in strong influences of iTBS on the MEP_MT1 mV_ amplitudes at different time points. The main effect of iTBS was significant (*F* (3, 57) = 126.851, *P* < 0.001), corresponding to the distinct difference of the real EA arms in Figure 2. Nonetheless, in the sham EA condition, there was no discerning iTBS × EA interaction (*F* (2.274, 43.206) = 0.186, *P* = 0.856) or main effect of iTBS (*F* (2.274, 43.206) = 0.023, *P* = 0.881). Thus, with the absence of EA, iTBS did not have distinct influence on the MEP_MT1 mV_ amplitudes at the time points. As shown in Figure 2, the sham EA arms does not differ significantly. The main effect of time was not significant (*F* (2.274, 43.206) = 1.199, *P* = 0.318), so the MEP_MT1 mV_ amplitude did not vary with time points.

Table 3 shows the Bonferroni-corrected pairwise comparison results of LOG MEP_MT1 mV_ amplitude at different time points of the sham-iTBS-real-EA arm. MEP_MT1 mV_ amplitude had a significant reduction at T0 and T20 compared to baseline (T0-baseline = −0.181, *P* = 0.001; T20-baseline = −0.121, *P* = 0.006), indicating that the condition of sham iTBS and real EA suppressed the MEP_MT1 mV_ amplitude significantly.  Table 4 shows the Bonferroni-corrected pairwise comparison results of LOG MEP_MT1 mV_ amplitude at different time points of the real-iTBS-real-EA arm. MEP_MT1 mV_ amplitude had a significant improvement at T0, T20, and T40 compared to baseline (T0-baseline = 0.141, *P* < 0.001; T20-baseline = 0.155, *P* = 0.001; T40-baseline = 0.112, *P* = 0.021), indicating that the condition of sham iTBS and real EA improved the MEP_MT1 mV_ amplitude significantly.

2-way ANOVAs with main factors of EA and time in the real iTBS and sham iTBS conditions were performed as well. Whether iTBS was sham or real, there was significant interaction between EA and time (sham iTBS: *F* (3, 57) = 5.658, *P* = 0.002, real iTBS: *F* (3, 57) = 5.342, *P* = 0.003).

In all the above experiments, there were no statistically significant differences in SICI, ICF, CMCT, and CSP between groups and between time points.

In summary, with real iTBS and real EA, the MEP_MT1 mV_ amplitude was significantly improved and lasted for 40 minutes after the intervention. On the contrary, with sham iTBS and real EA, MEP_MT1 mV_ amplitude was significantly suppressed and gradually returned to the pre-intervention level 40 minutes after the intervention. And when EA was sham, no matter whether iTBS was real or sham, there was no significant impact on MEP_MT1 mV_. And throughout the experiment, there were no statistically significant differences in SICI, ICF, CMCT, and CSP between groups and between various time points.

## 4. Discussion

In our study, we applied EA immediately after the end of iTBS on Hegu (LI4), Quchi (LI11), Zusanli (ST36), and Yanglingquan (GB34), which are four acupuncture points that are most commonly used in the rehabilitation of stroke exercise in the clinic. The results indicated that iTBS combined with EA can significantly increase the excitability of the motor cortex for 40 minutes after the end of the intervention, while applying EA alone will inhibit the excitability of the motor cortex. When the EA is sham, it does not cause changes in cortical excitability. So, this study demonstrates that the interaction of EA with iTBS increases cortical spinal cord excitability and the effect of this increase is extended.

### 4.1. The Effect of EA on Cortical Spinal Excitability

In our study, a sham acupuncture that did not invade the body was used as a control. It was found that the EA had an inhibitory effect on MEP amplitude but had no significant effect on SICI, ICF, etc. In former studies of Hegu (LI4) and Tiaokou (ST38) [13, 14], the researchers used real acupuncture at nonacupoint as a control group, and inhibition of motor cortex excitement was observed in experimental group and changes in MEP amplitude were also observed in control group. Zunhammer et al. [15] used the sham acupuncture without skin insertion as a control for the first time and reported that acupuncture at Yanglingquan (GB34) caused RMT elevation in the contralateral hemisphere, but AMT, SICI, and ICF had no significant variations. The results of the above studies are consistent with the results of our study. However, a double-blind, sham-controlled study showed that acupuncture at Hegu (LI4) did not cause changes in excitability of the motor cortex [24]. In general, acupuncture can cause an inhibition in excitability of the contralateral cerebral cortex, which is consistent with the results of this study. At present, there are relatively little studies with TMS on the mechanism of acupuncture to evaluate the excitability of the motor cortex, and the research qualities are relatively low, which limits the research progress of the acupuncture mechanism to some extent.

### 4.2. After-Effect of iTBS

There have been a lot of previous studies revealing that iTBS could improve the cortex excitability of healthy subjects, and the effect could last for 20–40 min. ITBS could effectively help the recovery of dyskinesia in stroke patients [1, 25, 26]. In our study, no changes in cortical excitability were observed after real iTBS + sham EA intervention. We speculate that this was because the iTBS intervention was followed immediately by the 20 min sham EA intervention before any MEP measurement. Therefore, no significant after-effect of iTBS was observed after 20 minutes of sham EA. In the real iTBS + real EA condition, the MEP amplitude increased significantly after the intervention, lasting more than 40 minutes, which was never observed in previous studies. Although the mechanisms involved in promoting neural plasticity are currently not fully understood, the well-defined forms of neuroplasticity include long-term potentiation (LTP) and long-term depression (LTD). Noninvasive brain stimulation (NIBS) technology can induce and measure LTP-and-LTD-like changes in the human brain [27], while iTBS improves cortical excitability by inducing LTP-like plasticity. Previous studies have shown that iTBS induces LTP-like plasticity by affecting N-methyl-D-aspartate receptor (NMDA-R [28]) and changing the expression of some proteins including zif268 [29] and calcium-binding protein [30], thereby enhancing the excitability of the motor cortex.

### 4.3. Possible Mechanism of Real iTBS + Real EA Intervention

Experiments on animals revealed that acupuncture can induce neurogenesis, synaptogenesis, and alterations of synaptic efficiency [31]. But unfortunately, the current number of studies on the effects of acupuncture on brain plasticity in healthy people is very limited, which significantly limited people's understanding of the influence of acupuncture on brain plasticity. After acupuncture needles penetrate the skin, they first activate the afferent fibers of the peripheral nerves, producing a feeling called “Deqi,” which is a feeling of numbness, filling, and pain [32, 33]. Through this strong sensory stimulation, acupuncture or electroacupuncture can promote the release of certain neurotransmitters in the central nervous system, such as norepinephrine, glutamic acid, dopamine, etc., thereby activating related receptors and downstream signalling pathways [34]. Therefore, it is speculated that regulation of the neurotransmitter system may be one of the potential mechanisms for acupuncture to regulate neural plasticity.

The results of our study showed that the application of EA immediately after the end of iTBS stimulation can significantly improve the excitability of the motor cortex. In addition to the possibilities mentioned above, the mechanism of this phenomenon may also be related to the priming effect. The priming effect is based on a concept of meta-plasticity, the plasticity process of synaptic plasticity, which refers to the change in the threshold required for subsequent synaptic excitability changes due to some previous activity of the brain network [35]. NIBS can have an effect on the meta-plasticity of young healthy subjects, and this change in neural plasticity is stronger, longer lasting, and more stable [36]. The change in one type of NIBS effect caused by the priming of another type of NIBS is not only related to the type of stimulation of the NIBS, but also closely related to the time interval between the two NIBS stimulation protocols [37]. However, most of the current studies on meta-plasticity focus on two different NIBS protocols acting on the same cortical region, exploring the priming effect of one NIBS on another. There are few studies [38–40] on the initiation of a single NIBS protocol combined with other interventions. In future research, how to combine NIBS with acupuncture and use meta-plasticity to improve functional recovery of stroke should be paid more attention to.

In summary, the underlying mechanisms of acupuncture and iTBS regulation of motor cortex excitability may have similar neural pathways and molecular biological mechanisms and in some special cases produce a mutually reinforcing effect, thereby inducing stronger, more persistent cortical excitability changes. Unfortunately, there are no relevant animal experiments to show how the two have produced this interaction. Future research should focus more on basic research that can reveal this phenomenon.

## 5. Limitations

In this study, only SICI of 2 ms interval and ICF of 10 ms interval were selected as indicators to evaluate neural circuits, but the changes of SICI and ICF indicators in other intervals were not tested, which may have missed some potential mechanisms, which will be further supplemented by future studies.

The use of TMS-MEP and pp-TMS to study the central effect of acupuncture is still a small number of reports, and there are no high-quality studies, or large sample clinical trials to verify. This will be the focus of future research.

## Figures and Tables

**Table 1:** Physiological indexes at baseline.

iTBS	EA	RMT (% MSO)	AMT (% MSO)	LOG MEP_MT1 mV_ (log (uV))
Sham	Sham	37.0 ± 4.28	—	3.05 ± 0.07
	Real	37.1 ± 4.66	—	3.05 ± 0.05

Real	Sham	37.1 ± 4.73	27.3 ± 2.58	3.03 ± 0.05
	Real	37.4 ± 4.92	27.5 ± 2.24	3.05 ± 0.07

MSO, mean stimulator output. Data format is mean ± standard error.

**Table 2:** ANOVA results.

ANOVA	Main factors	Degrees of freedom; error	Effects	*F*	*P*
3-way	iTBS × EA × Time	3, 57	*I* × *E* × *T*	11.02	<0.001
			*I*	62.61	<0.001
			*E*	0.084	0.774
			*T*	0.659	0.581

2-way	iTBS × Time				
	Sham EA	2.27, 43.21	*I* × *T*	0.186	0.856
			*I*	0.023	0.881
			*T*	1.199	0.318
	Real EA	3, 57	*I* × *T*	23.48	<0.001
			*I*	126.9	<0.001
			*T*	1.019	0.391

2-way	EA × Time				
	Sham iTBS	3, 57	*E* × *T*	5.658	0.002
			*E*	18.00	<0.001
			*T*	5.669	0.002
	Real iTBS	3, 57	*E* × *T*	5.342	0.003
			*E*	28.24	<0.001
			*T*	7.573	<0.001

**Table 3:** Pairwise comparison results of the sham-iTBS-real-EA arm.

	LOG MEP_MT1 mV_	
	Difference	*P*
T0-baseline	0.141	<0.001
T20-baseline	0.155	0.001
T40-baseline	0.112	0.021
T20-T0	0.014	1.00
T40-T0	−0.029	1.00
T40-T20	−0.043	1.00

**Table 4:** Pairwise comparison results of the real-iTBS-real-EA arm.

	LOG MEP_MT1 mV_	
	Difference	*P*
T0-baseline	−0.181	0.001
T20-baseline	−0.121	0.006
T40-baseline	−0.066	0.283
T20-T0	0.060	0.971
T40-T0	0.115	0.106
T40-T20	0.055	0.895

## Data Availability

Data are available on http://www.chictr.org.cn/showproj.aspx?proj=42076.
